# Evolution of eumetazoan nervous systems: insights from cnidarians

**DOI:** 10.1098/rstb.2015.0065

**Published:** 2015-12-19

**Authors:** Iva Kelava, Fabian Rentzsch, Ulrich Technau

**Affiliations:** 1 Department of Molecular Evolution and Development, Faculty of Life Sciences, University of Vienna, Althanstrasse 14, 1090 Vienna, Austria; 2 Sars Centre, Sars International Centre for Marine Molecular Biology, Thormøhlensgt. 55, 5008 Bergen, Norway

**Keywords:** Cnidaria, nervous systems, neurogenesis, evolution, development

## Abstract

Cnidarians, the sister group to bilaterians, have a simple diffuse nervous system. This morphological simplicity and their phylogenetic position make them a crucial group in the study of the evolution of the nervous system. The development of their nervous systems is of particular interest, as by uncovering the genetic programme that underlies it, and comparing it with the bilaterian developmental programme, it is possible to make assumptions about the genes and processes involved in the development of ancestral nervous systems. Recent advances in sequencing methods, genetic interference techniques and transgenic technology have enabled us to get a first glimpse into the molecular network underlying the development of a cnidarian nervous system—in particular the nervous system of the anthozoan *Nematostella vectensis*. It appears that much of the genetic network of the nervous system development is partly conserved between cnidarians and bilaterians, with Wnt and bone morphogenetic protein (BMP) signalling, and *Sox* genes playing a crucial part in the differentiation of neurons. However, cnidarians possess some specific characteristics, and further studies are necessary to elucidate the full regulatory network. The work on cnidarian neurogenesis further accentuates the need to study non-model organisms in order to gain insights into processes that shaped present-day lineages during the course of evolution.

## 1. Why study cnidarian nervous systems?

**Figure 1.**
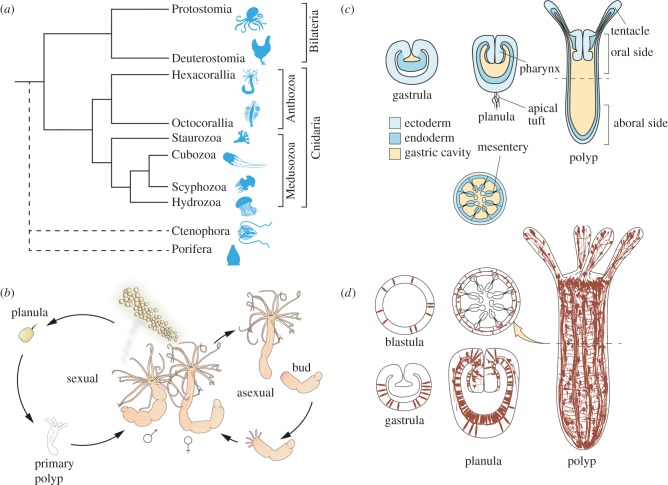
(*a*) Phylogenetic relationships of cnidarians (after [4]). As the phylogenetic position of ctenophores and sponges is still not completely resolved, their lineages are marked with a dashed line [3,5–8]. The length of branches is for illustrative purposes only and does not represent time of divergence. (*b*) Life cycle of *Nematostella vectensis*, with both sexual and asexual reproduction. (*c*) Schematic of the body plan of *N. vectensis* throughout development. The lower panel represents a section through the polyp at the dashed line. (*d*) Schematic of the nervous system of *N. vectensis* throughout development. Neurons are depicted in brown. The schematic is based on several studies (see below), but it probably does not represent the whole neuronal population. All stages are showed as sections, except for the primary polyp. The section through the polyp (at the dashed line) shows the distribution of neurons in different layers.

**Figure 2.**
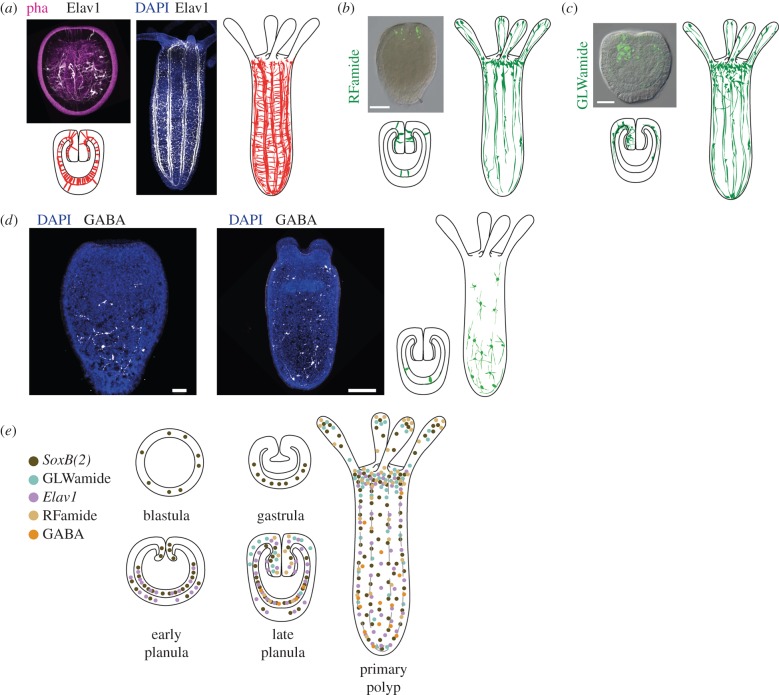
(*a*) Distribution of *Elav1*-positive neurons in planula (left) and primary polyp (right). In the microscopic images (taken from reference [27]), *Elav1*-positive neurons are in white, phalloiding is in purple, and DAPI is in blue. In the schematic, *Elav1*-positive neurons are in red. (*b*) Distribution of RFamide-positive neurons in planula (left) and primary polyp (right). The inset is from reference [28]. RFamide-positive neurons are in green. (*c*) Distribution of GLWamide-positive neurons in planula (left) and primary polyp (right). The insert is from reference [28]. GLWamide-positive neurons are in green. (*d*) Distribution of GABA-positive neurons in planula larva and the primary polyp (GABA, white; DAPI, blue). The original data images are maximum projections of 20–30 single confocal images. (*e*) Distribution of different neuronal subpopulations during the development of *N. vectensis*. The schematic is based on the results of immunostaining with the antibody against the neuropeptide (GLWamide (turquoise) [28], RFamide (beige) [26,28], GABA (orange) (I.K. and U.T. 2015, unpublished data), or on the analysis of transgenic animals in which a fluorophore is under the control of the gene of interest promoter (*SoxB(2)* (brown) [29], *Elav1* (purple) [27]). Scale bars, (*b,c*) 100 µm; (*d*) 50 µm (planula), 100 µm (primary polyp).

**Figure 3.**
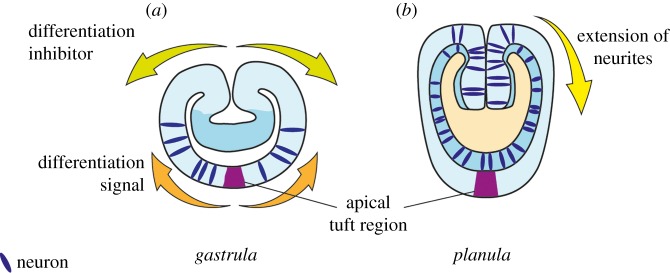
Early neurogenesis switch in *N. vectensis*. During very early development (blastula to ea rly gastrula; (*a*)), individual neurons start appearing in the aboral half, yet excluding future apical organ at the aboral pole. The location and morphology of the cells speak in favour of neuronal determination *in situ*, either guided by stochastic processes, or by the action of a yet-unknown gradient. Neurogenesis during later stages of embryonic development (*b*) is still poorly understood, but seems to have a more localized character. In the planula, many neurons are now born at the oral side and in the endoderm. It is still unclear whether and to what extent neuronal precursors migrate towards the aboral side of the embryo.

With the exception of Placozoa and Porifera, the nervous system is a defining characteristic of Metazoa, and its appearance was probably a crucial determinant in their diversification and their capability to conquer almost all ecological niches. Although the nervous system has been at the focus of attention for many years, and many aspects of its development and physiology are well understood, the knowledge about its evolutionary origins is still in its infancy. Most research on nervous systems has been carried out on standard model organisms, but their restricted phylogenetic representation makes it difficult to propose viable theories about the ancestral morphology and development of the nervous system. While the contentious phylogenetic positions of Porifera, Placozoa and Ctenophora are impacting on scenarios of the evolution of the nervous system (see also [1]), the Cnidaria have a robust position as a sister group to the Bilateria (figure 1*a*, [2,3]). Hence, the Cnidaria and the comparison with Bilateria are crucial for the reconstruction of a cnidarian–bilaterian ancestor and our understanding of the evolution of eumetazoan nervous systems. The cnidarians are divided into two major groups, the Anthozoa, consisting of Hexacorallia and Octocorallia, and the Medusozoa, which comprise Hydrozoa, Scyphozoa, Cubozoa and Staurozoa (figure 1*a*; [9]). The relatively simple morphology, underlined by an intricate gene repertoire, makes cnidarians an ideal system for studying the developmental and cellular processes that (i) led to the emergence of the nervous system and (ii) were involved in the adaptation of nervous systems to different environments and over long periods of time.

Until recently, most of our knowledge on the development of cnidarian nervous systems came from *Hydra*. *Hydra* has been very helpful for getting insights into the mechanisms of neuronal differentiation during regeneration and homeostasis in the polyp (see below), however, it mainly propagates asexually by budding. The embryonic development occurs infrequently and is relatively derived, which makes it difficult to investigate and analyse the cellular and molecular differentiation processes during the initial formation of the nervous system.

Other species of the Medusozoa typically have a more complex life cycle, which involves a pelagic medusa stage and a sessile polyp stage. Medusae generally exhibit a more complex nervous system, with neural rings and eyes that are organized in rhopalia and statocysts. Processing and integration of information has been described in rhopalia [10,11], and the high concentration of neurites in the ecto- and endodermal nerve rings at the medusa bell appears to have a function in controlling swimming behaviour [12]. The more complex repertoire of sensory organs in the medusae allows for a more elaborate set of behaviours than found in the purely benthic polyps, and nerve rings and rhopalia may represent an independently evolved form of nervous system centralization [13].

In this review, we discuss cnidarian nervous systems with an emphasis on the recent findings in anthozoan starlet sea anemone *Nematostella vectensis* (figure 1*b*), because this system is amenable to functional studies investigating neurogenesis during embryogenesis. *Nematostella* became an important model system among cnidarians in the past decade [4,14–16]. This brackish water organism has been put forward among other anthozoans owing to its accessibility and amenability for experimental research. It is readily kept under laboratory conditions, spawning can be induced reproducibly, the genome has been sequenced, and gene knockdown methods and stable transgenics have been established, which were particularly insightful for our current understanding of neuronal development [17–19] (for review, see [15]). *Nematostella* has a surprisingly complex genome, including all major signalling pathways and most transcription factor families [18,20–24].

## 2. Structure of the *Nematostella* nervous system

The nervous system of *Nematostella*, as of other cnidarians, is comprised of two interconnected neuronal networks, one in the ectoderm and one in the endoderm. The principal cell types of cnidarian nervous systems are sensory cells, ganglion cells (the morphological equivalent of interneurons) and cnidocytes (stinging cells). Molecular analyses have revealed that the neuronal networks and the three main classes of neural cells comprise several subpopulations of neurons, which are marked, e.g. by the expression of different neuropeptides, and which can have different distributions along the body.

The search for a pan-neuronal marker for cnidarian neurons has been more difficult than expected. One of the candidate genes for a pan-neuronal marker is the homolog of *Elav1*, coding for an RNA-binding protein involved in neuronal differentiation [25]. In *Nematostella*, it indeed marks a large neuronal subpopulation [26,27], but is not a pan-neuronal marker (figure 2*a,e*), as it is part of a larger population marked by *SoxB(2)* [29] (see below).

In the polyp, *N. vectensis*, the neuron density appears to be higher in the oral half of the animal, but previous suggestions of an oral nerve ring [26] could not be confirmed. *Elav1*-positive neurons form networks both in the ectoderm and in the endoderm (figure 2*a,d*). In the endoderm, however, many neurons follow the parietal muscles on either side of the eight mesenteries, forming prominent longitudinal tracts [27]. These tracts are connected via anastomoses of single neurons.

Two smaller neuronal subpopulations expressing specific neuropeptides, RFamide and GLWamide [30,31], are primarily found in the oral half of the young polyp. Both of them are found in all cnidarians and many bilaterians examined so far [26,28,32–34] (figure 2*b,c,e*). Individual RFamide-positive neurons appear in the tentacles (figure 2*b,e*) and may, as in *Hydra* and other hydrozoans, have a role in ectodermal sensory neurons.

The GLWamide-positive neuronal subpopulation has recently been shown to have a very interesting development. This population appears 2–3 days post fertilization (dpf), but, unlike the RFamide-positive neurons, the GLWamide-positive neurons initially appear on the one side of the developing pharynx in the planula [28]. Later, more GLWamide neurons are added in a radially symmetric pattern, similar to RFamide neurons, and the asymmetrical distribution of GLWamide-positive neurons becomes undetectable (figure 2*c,e*). However, we still have no insights into which special behaviour or physiological processes this (and maybe other) asymmetrical neuronal subpopulations might be involved in. It has been reported that at 4 dpf these subpopulations make up around 8% of the *Elav1*-positive neurons [28]. However, because *Elav1* is not a pan-neuronal marker, the fraction of RFamide or GLWamide neurons of all neurons is unclear.

Both these neuropeptides mark neurons, which appear in both endoderm and ectoderm, but layer-specific neuronal populations have not yet been described. Recently, we discovered that the neurotransmitter gamma amino butyric acid (GABA) marks a population of neurons that is present only in the endoderm, with the tendency to be more concentrated in the aboral region (figure 2*d,e*; I.K. and U.T. 2015, unpublished data). Interestingly, the GABA-neurons do not appear to form connections to each other and seem embedded as individual neurons in the nervous system, raising questions about their role. The confined expression of individual neuronal markers reveals a hidden complexity of the *Nematostella* nervous system, with neuronal subpopulations that might be dedicated to different processes and/or behaviours.

When comparing *Nematostella* with polyps of other cnidarian species, we can see notable differences in the structure of neuronal subpopulations. For instance, in *Hydra*, RFamide marks mostly ectodermal sensory neurons of the hypostome and tentacles, but also ectodermal ganglion neurons of the peduncle, although they might also have at least a propriosensory function, because ultrastructural studies showed that they contain cilia [35]. In the planula larva of *Clava multicornis*, a hydrozoan, RFamide-positive neurons accumulate at the anterior end [36]. The difference between *Nematostella* and these species may reflect different constraints in their biology and shows that evolutionary interpretations of neuronal patterns have to be taken with caution.

Bioinformatic analysis of the *Nematostella* genome has shown that a large number of genes associated with chemical neurotransmission are present [37]. Based on these data, we can conclude that the number of neuronal subpopulations in *Nematostella* must be larger than the several described ones (see above), maybe numbering in dozens. However, in the absence of a pan-neuronal marker, limited number of antibodies and the lack of possibilities for double and triple stainings, it will be difficult to conclude which fraction of neurons express two or more neuropeptides. Also, one has to take into account the discrepancy of bioinformatic data with the empirical data (lack of serotonin orthologues in reference [37], with serotonin immunostaining in reference [26] in *Nematostella*). In order to tackle this problem, it is necessary to develop antibodies against cnidarian neurotransmitters and improve the immunostaining protocols.

## 3. Establishment of the nervous system in *Nematostella vectensis*

In most bilaterians, the neurogenic potential is unequally distributed in the ectodermal tissue. While sensory neurons can often be generated throughout most of the ectoderm, interneurons are typically generated only in the so-called neuroectoderm, the territory from which the central nervous system (CNS) develops. With some exceptions (e.g. hemichordates, acoel worms and flatworms), the specification of the neuroectoderm and CNS is a result of the formation of the dorsoventral (DV) axis by a gradient of BMP signalling [38]. While most cnidarians are considered radially symmetric, anthozoans form a second body axis, the directive axis, which depends on a gradient of BMP signalling [21,39,40]. However, this BMP signalling gradient has been detected only considerably later (at gastrula stage, [39,40]) than the occurrence of the first neural progenitor cells (NPCs; at mid-blastula stage [29]) and early neural differentiation is not biased along the directive axis [26,27,29,41]. Interestingly, the previously mentioned asymmetric distribution of early GLWamide-positive neurons (see above), together with the RFamide-positive population, depends on BMP signalling along the directive axis [28].

Recent observations suggest that in *Nematostella* neurogenesis commences at mid-to-late blastula stage in an aboral territory that spans approximately 75% of the body length (figure 3*a*). At early gastrulation, the oral cap is devoid of differentiating neurons, whereas after gastrulation, more neurons—including some specific subpopulations (RFamide, GLWamide neurons)—are born in the oral half (in and around the pharynx) and in the endoderm [26–28] (figure 3*b*). Notably, the most aboral region, the ‘apical organ’, often referred to as a sensory centre, remains free of *Elav1* and *SoxB(2)* neural cell bodies [27,29]. It is unclear which signals prevent the early neurogenesis at the oral domain. Because the blastopore expresses various *Wnt* genes, and the oral–aboral axis of *Nematostella* is patterned by Wnt signalling [42,43], Wnt signalling might be involved in suppressing early neuronal differentiation at the oral pole. However, in contrast to this idea, recent work showed that manipulation of the Wnt pathway affects the development of oral RFamide and *Elav1* neurons [28], suggesting a conserved role for Wnt signalling in promoting neurogenesis. This switch from aboral to more orally located neurogenesis during embryonic development might also indicate a shift from early on-site differentiation of neurons, to a somewhat more restricted neurogenic field. It would be interesting to investigate the potential migration patterns that the neural progenitors and/or neurons, born in this more restricted area, undergo, in order to establish the nervous system of an adult, both in the ecto- and endoderm.

While in bilaterians neurons originate from the ectoderm, in *Nematostella*, both ectoderm and endoderm appear to be capable of producing neurons [27]. Endodermal neurons appear shortly after completion of gastrulation. By using transplantation experiments between transgenic *Elav1::memOrange* and wild-type embryos, Nakanishi *et al.* [27] have shown that the endoderm can produce neurons independently of the ectoderm. Whether the underlying genetic programme and developmental processes are the same in these two germ layers remains to be elucidated. Because nematocytes differentiate only in ectodermal tissue, one would expect distinct molecular mechanisms that ensure nematocyte and neuronal differentiation in the ectoderm, but neuronal differentiation only in the endoderm.

The differentiation of neurons as assessed by the formation of basal neurites begins at late gastrula stage and becomes more prominent at early planula stage. Analysis of the *SoxB(2)::mOrange* transgenic line, which broadly labels neural progenitors and their progeny, showed that neurites can extend in any direction from the onset of differentiation [29]. Interestingly, *Elav1::mOrange-*positive sensory cells, which constitute a subset of the *SoxB(2)::mOrange* cells, predominantly project in an aboral direction at early- and mid-planula stage. Later-born *Elav1* neurons, however, preferentially project in transverse orientation. This is paralleled by the development of the mesenteries, and soon neuronal tracts run along the parietal muscle in the mesentery, with individual neurons situated in between and connecting them [27]. This change in the neurite projection pattern may indicate chemical cues that turn on and off during development in order to correctly orient the projections of neural subpopulations in the developing nervous system; however, they have not yet been identified. In fact, the expression patterns of candidate guidance molecules, such as Netrin or RGM, do not obviously relate to the observed changes in neurite projections of *Elav1*-positive neurons [24,39].

The development of the nervous system in *Nematostella* displays some striking differences to that in *Hydra* and other hydrozoans such as *Clytia hemisphaerica* and *Hydractinia echinata*. In these cnidarians, neurons, as well as nematocytes (cnidocytes), differentiate from multipotent interstitial stem cells (i-cells). i-cells predominantly reside between the ectodermal epithelial cells of the body column. Interestingly, the distribution of i-cells is virtually complementary to the density of neurons, which are highest at both extremities, i.e. in hypostome, tentacles and peduncle. i-cells become committed to become neurons either stochastically or by unknown signals. Neuronal progenitors then migrate orally or aborally to the site of differentiation, where they undergo a final mitosis and differentiate—probably by local cues—to a specific neuronal phenotype [44,45]. However, i-cells have only been found in hydrozoans and therefore are considered a specific feature of hydrozoans. In other cnidarians, e.g. the scyphozoan *Aurelia aurita*, neurons likely arise from epithelial cells or intermediate progenitors, more akin to the situation in *Nematostella* [46]. This variability further emphasizes the need to compare several species of one clade.

## 4. Developmental genetics of *Nematostella* neurons

The publication of several cnidarian genomes has shown that much of the molecular architecture underlying neurogenesis and neuron functioning is conserved between bilaterians and cnidarians [17,47–51]. In bilaterians, neurons are born from specialized cell populations, termed NPCs, which arise in an area of the ectoderm dedicated to developing the CNS. With cnidarians lacking a centralized system, and having both ectodermal and endodermal neurogenesis, it has been questioned whether this conserved molecular toolkit is employed in the same way.

Recent research on Wnt and BMP signalling during embryonic neurogenesis in *Nematostella* gives us some insight into the involvement of these conserved pathways in cnidarian neurogenesis [28]. The Wnt/β-catenin pathway is involved in neural patterning and neurogenesis in bilaterians [52,53]. Use of a β-catenin signalling inhibitor resulted in a severe reduction of RFamide, GLWamide and *Elav1::mOrange* neurons at planula stage, whereas ectopic activation of β-catenin increased the number of these neurons [28]. These observations suggest that β-catenin can positively regulate neural development in *Nematostella*. However, because the oral Wnt signalling centre of the blastopore is devoid of early neurogenesis, it is not clear whether Wnt/β-catenin signalling has a direct role in early neurogenesis or a general positive function in establishing neurogenic potential. Surprisingly, while in flies and vertebrates the gradient of BMP signalling along the DV axis has an anti-neuralizing effect and localizes the CNS [38], in *Nematostella*, BMP signalling appears to have no effect on neuronal differentiation at an early phase, but a proneural function in the later phase of embryonic neurogenesis [28]. Future research also on other bilaterian phyla will reveal whether Wnt signalling or BMP signalling (or both) has an ancestral role in neurogenesis.

Interesting insights into the conservation of regional patterning came from the analysis of the bilaterian head patterning genes *six3/6*, *FoxQ2a* and *irx*, which are early anterior brain markers. Strikingly, *six3/6*, *FoxQ2a* and *irx* are actually expressed at the aboral end of the *Nematostella* planula [54], suggesting a stunning conservation of regional patterning genes. Knockdown of *Nematostella six3/6* reduced the number of *DmrtB*-expressing aboral neurons, but did not affect the expression of the broader neural marker RFamide, suggesting that the effect on the aboral neurons is rather a consequence of the mis-specification of the aboral domain. These observations are similar to loss-of-function studies in sea urchin and the beetle *Tribolium castaneum* [55,56], but different from the situation in vertebrates, where *six3/6* is crucial for anterior brain development. Thus, general axial patterning genes might have been coopted for the induction of the anterior CNS in the vertebrate lineage.

The genetic network underlying the transition from the NPC to the post-mitotic neuron has been extensively studied in *Drosophila* [57] and mammals, especially mouse [58]. The determination of NPCs from the epithelial layer represents a textbook example of the lateral inhibition by the Notch/Delta system [59]. In a previously designated neuroepithelial field, NPCs, which are destined to become neurons, are singled out by the interaction of receptors, and by differential expression of the neurogenic programme in individual cells. The cells, which remain dividing progenitors, are inhibited from expressing this programme for the moment. It appears that, in *Nematostella*, the Notch/Delta system is also involved in determining the fate of neural lineage cells. By using pharmacological treatments with the *γ*-secretase inhibitor DAPT, it was shown that the Notch/Delta signalling influences the expression of neurogenic markers [60], e.g. the *achaete-scute* homologue (*AshA*) among others [61].

More surprising was the finding that the Notch/Delta function in neurogenesis is not conducted through the canonical pathway, i.e. involving *suppressor of hairy* (*Su(H)*), but a non-canonical, yet unidentified route [61]. This difference might indicate that the Notch pathway had a more general role in cell differentiation in the ancestor of cnidarians and bilaterians [62], and was coopted in slightly different ways in the neurogenic pathways of both lineages. Another explanation is that the non-canonical Notch signalling is the ancestral form of this signalling pathway. This is based on the fact that only bilaterians have the full complement of the Notch/Delta pathway [63]. However, because two key elements of canonical Notch signalling, *Su(H)* and *mastermind*, are present in *Nematostella*, this hypothesis still awaits confirmation through data from other, non-bilaterian phyla.

Downstream of Notch/Delta signalling, a specific set of proneural genes of the bHLH transcription factors become activated, in particular the *achaete-scute* (*Ash*) and *atonal* (*ato*) gene family, which regulate the transition of the progenitor cell into a neuron. At least one, *AshA*, is expressed in single cells of the aboral half of the early embryo and is directly involved in neurogenesis: knockdown leads to loss of specific neuronal markers, overexpression increases the number of RFamide+ and *Elav1*-precursor cells in the aboral half [41]. The data suggest that *AshA* does not have a pan-neuronal role, which would also fit the model in which *Ash* and *ato* promote neurogenesis of distinct neuronal populations [64], as in bilaterians. However, as the data on the members of the *ato* family are still scarce, owing to their unresolved phylogeny [65], this idea cannot be yet confirmed. The expression patterns of several *ato* genes coincide with the expression patterns of *AshB* and *SoxB2*, a gene also involved in neurogenesis in Bilateria [28]. Interestingly, one of the *ato* genes, *Arp6*, is expressed asymmetrically in the developing embryo and functional analysis suggests that it regulates the asymmetric distribution of GLWamide-positive neurons. Notably, in hydra, chemical inhibition of Notch signalling suggests a role in boundary formation during detachment of the bud and in tentacle formation during regeneration [66], thus, fundamentally different processes from neuronal differentiation. It is possible that Notch signalling has distinct roles in embryogenesis and adult polyps.

In mammals, neural progenitors still have the capability to divide and produce either other types of progenitors or different subpopulations of neurons. *Pax6* and *Sox2* are markers of these intermediate progenitors in the developing mammalian brain [58]. It is still not clear whether a neural progenitor population similar to this one exists in cnidarians. Until recently, it was also unclear whether cnidocytes and neurons, both members of the neuronal lineage, come from the same populations of progenitors. Here, Sox proteins might be key to this question. Sox proteins are indispensable in the determination and maintenance of embryonic stem cells in mammals, and later, during brain development, in the population of NPCs [67]. Members of SoxB1 and SoxB2 subgroups are especially important during neurogenesis [68,69]. Of the 14 *Sox* genes present in *Nematostella* and *Hydra* [17,24,47,70], one gene, *SoxB(2)* (termed *SoxB2* in reference [24] and *SoxBa* in reference [71]), is expressed in single cells during gastrulation, consistent with a role in neuronal differentiation [24]. Using a *SoxB(2)* transgenic reporter line, Richards and Rentzsch showed that *SoxB(2)* marks a population of cells that gives rise to ganglion and sensory neurons and cnidocytes, thus representing a general neural progenitor population [29]. The knockdown of *SoxB(2)* strongly reduces the production of neurons and cnidocytes. Thus, this gene appears necessary for the differentiation of both cell types. The tracing of EdU-labelled dividing *SoxB(2)*-positive cells suggested that daughter cells of one neural progenitor in *Nematostella* can have different cell cycle characteristics [29], a feature that is reminiscent of asymmetric cell fate in *Drosophila* and mammals [72]. It also shows that this *Sox* gene has a conserved role in neurogenesis in cnidarians and bilaterians. In addition to *SoxB(2)*, as mentioned above, another *SoxB2* gene is involved in patterning the oral nervous system [28]. Taken together, these data strongly suggest that some key aspects of the neurogenic programme are conserved between cnidarians and bilaterians. Interestingly, *Sox* genes are also expressed in putative progenitors that give rise to neurosensory cells in the ctenophore *Pleurobrachia pileus* [73]. This further confirms the ancestral role that *Sox* genes have in the development and evolution of the nervous systems, but also brings into question the independent origin of nervous systems in ctenophores [5,74,75].

## 5. Evolutionary context

Taking into account more than 500 million years of independent evolution of the bilaterian and cnidarian lineage [76], surprisingly, many elements of the developmental neuronal network and genes governing neuronal structure and function are conserved. We conclude that the last common ancestor of Bilateria and Cnidaria was an animal with a well-established nervous system, in which neurons were born out of epithelial cells, which were singled out to become neurons by a cell-determining system (e.g. the Notch/Delta system) [61] and this mechanism was inherited from the common ancestor of sponges and eumetazoans [77], which may or may not have had a nervous system. These epithelial cells most probably underwent an asymmetric division, to produce a differentiated cell—a neuron, and presumably another epithelial cell or a neural progenitor. What also seems to be conserved is the early proneural gene network, which is involved in the production of neurons during the embryonic stages.

It has been shown that members of the *Sox* family of genes are involved in the patterning of the neural field and the production of the components of the neural lineage [28,29]. After the specification of the cell as a neural progenitor, other downstream proneural genes finish the differentiation process of the progenitor into a neuron [41]. More studies are needed in order to fully reconstruct the basic genetic network underlying cnidarian neurogenesis, but some obvious candidates exist. Members of the *Pax* family of transcription factors represent an interesting starting point, as they are crucial for mammalian neurogenesis (especially eye development), with *Pax6* being one of the main markers of neural progenitors. The findings that jellyfish *PaxB* gene is involved in the eye development of the cubomedusa *Tripedalia cystophora* and that *PaxB* can rescue a *Drosophila* eye mutant [78] suggests a conserved role of *PaxB* in neuronal development. In line with this, *Nematostella PaxA* and *PaxB* are expressed in single cells, reminiscent of a pattern present in progenitors and/or neurons [24]. The finding of asymmetrical divisions in the *SoxB(2)*-positive progenitors also invites the investigation of cell polarity proteins (e.g. Par3, Par6), and their role in the determination of cell fate. With the development of advanced *in vivo* imaging techniques, this problem becomes more accessible.

All of the previous studies spanning more than 150 years of cnidarian research points at the common origin of the nervous systems in Bilateria and Cnidaria. However, there are also marked differences. The curious aspect of endodermal neurogenesis existing in *Nematostella* presents a puzzle. Is neurogenesis in both germ layers an ancestral trait, and bilaterians have lost it or was it independently gained in the cnidarian lineage? Further detailed analyses of the processes governing neurogenesis in both germ layers, and potential comparison with genomic elements expressed in the bilaterian endoderm, are necessary to resolve this question. The cnidocyte, the cnidarian-specific cell type, is also a part of the neuronal lineage, as it seems that it stems from the same progenitor pool. Interestingly, cnidocytes express one of the three subfamilies of the ether-à-go-go (EAG) family of voltage-gated K^+^ channels [79]. However, its morphology and function are markedly different from any neuronal type in the rest of the animal kingdom. While our knowledge of the molecular basis of neuronal physiology is still scarce, there has been some recent progress. *Nematostella* has a significantly expanded set of 20 voltage-gated K^+^ channels of the shaker family, yet their function in *Nematostella* is still unclear [80]. Further, the diversification of the EAG family of voltage-gated K^+^ channels into Eag, Erg and Elk subfamilies occurred in the cnidarian/bilaterian ancestor after divergence from ctenophores. All three subfamilies seem to have at least partially conserved molecular functions, when tested *in vitro* [79,81]. An interesting case is the voltage-gated Na^+^ (Na_v_) channels, which are responsible for the action potential of neurons in bilaterians. Both cnidarian and bilaterian Na_v_ channels have evolved from an ancestral voltage-gated Ca^2+^ (Ca_v_) channel. However, the selectivity filter differs significantly in cnidarians and bilaterians, suggesting that a key component of neuronal physiology has evolved independently in these two lineages [82,83].

## 6. Outlook

Evolutionary developmental biology (evo-devo) has experienced a renaissance in the past 10–15 years. This is mostly owing to the development of new functional techniques and the advancement of sequencing methods. With this, scientists could return to using non-model organisms to answer questions about the evolutionary origin of pathways, cells, organs and whole systems. Cnidarians have been particularly interesting in the studies of germ layers and the nervous system. However, there are still many unanswered questions. One of the main puzzle pieces still missing is the molecular signature of the cnidarian neural progenitors and neurons, and how it relates to the bilaterian ones. This could be addressed by using a combination of transgenic animals and transcriptome sequencing, in order to decipher the molecular fingerprint of different neuronal populations. The cellular processes of the establishment and maintenance of the nervous system are also still largely unknown. Furthermore, the molecular and cellular basis of the dynamics and the physiology of the diffuse nervous system under conditions of homeostasis and growth are still not well understood. Applying new *in vivo* imaging techniques will allow us to track transgenic progenitor cells in the developing embryo and polyp to provide further insights into the formation and function of the cnidarian nervous system.
